# Dramatic age-related changes in nuclear and genome copy number in the nematode *Caenorhabditis elegans*

**DOI:** 10.1111/j.1474-9726.2007.00273.x

**Published:** 2007-04-01

**Authors:** Tamara R Golden, Kenneth B Beckman, Andreia H J Lee, Nancy Dudek, Alan Hubbard, Enrique Samper, Simon Melov

**Affiliations:** 1 Buck Institute for Age Research 8001 Redwood Boulevard, Novato, CA 94945, USA; 2 Center for Genetics, Children's Hospital Oakland Research Institute Oakland, CA 94609, USA; 3 Division of Environmental Health Sciences, School of Public Health, University of California Berkeley, CA, 94720, USA

**Keywords:** aging, *C. elegans*, genome, nematode, oldest old, variability

## Abstract

The nematode *Caenorhabditis elegans* has become one of the most widely used model systems for the study of aging, yet very little is known about how *C. elegans* age. The development of the worm, from egg to young adult has been completely mapped at the cellular level, but such detailed studies have not been extended throughout the adult lifespan. Numerous single gene mutations, drug treatments and environmental manipulations have been found to extend worm lifespan. To interpret the mechanism of action of such aging interventions, studies to characterize normal worm aging, similar to those used to study worm development are necessary. We have used 4′,6′-diamidino-2-phenylindole hydrochloride staining and quantitative polymerase chain reaction to investigate the integrity of nuclei and quantify the nuclear genome copy number of *C. elegans* with age. We report both systematic loss of nuclei or nuclear DNA, as well as dramatic age-related changes in nuclear genome copy number. These changes are delayed or attenuated in long-lived *daf-2* mutants. We propose that these changes are important pathobiological characteristics of aging nematodes.

## Introduction

Aging is generally defined and studied as a population phenomenon. Yet some aspects of aging, such as differences in individual lifespans, can only be understood through the study of individuals. An enigmatic aspect of aging is the heterogeneity in lifespan experienced by individuals of a synchronized population (Finch, 1990; Finch & Kirkwood, 2000). This heterogeneity, which can comprise up to 50% of the maximum lifespan of a species, is also observed in a clonal population of *Caenorhabditis elegans*, in which individuals are genetically identical and raised in an identical environment. The study of aging at the level of the individual organism is something for which *C. elegans* is well suited. In addition to being genetically identical, young adult worms are also essentially physically identical; studies of nematode development have mapped the cellular divisions and migrations leading to the final location of each of the 959 somatic nuclei in the young adult worm. However, such studies of worm morphology have not been applied to the remainder of the nematode lifespan.

Only recently have studies begun to examine the structural integrity of older adult worms to investigate the pathobiology of worm aging (Garigan *et al.*, 2002; Herndon *et al.*, 2002). Herndon *et al.* (2002) found evidence of sarcopenia in aging worms, whereas neurons appeared to remain intact throughout the nematode lifespan. This study also observed a stochastic element to aging; various cells and tissues deteriorated at different rates between individuals, and no single pathology or series of pathogenic changes characterized aging in all individuals.

Several mutations have been identified that extend the lifespan of *C. elegans* (Friedman & Johnson, 1988a, b; Johnson, 1990; Kenyon *et al.*, 1993; Kimura *et al.*, 1997; Lin *et al.*, 1997; Ogg *et al.*, 1997; Apfeld & Kenyon, 1999). The best-characterized single gene mutation that extends *C. elegans* lifespan is *daf-2*, which encodes an insulin-receptor homolog. Several mutations of *daf-2* have been isolated, which fit into two broad classes, class 1 and class 2 mutations (Gems *et al.*, 1998). In addition to other criteria, class 1 alleles have increased longevity, and lay a reduced number of unfertilized oocytes compared to wild-type worms, while class 2 alleles demonstrate these and additional traits, including reduced brood sizes, reduced adult motility, and production of late progeny (Gems *et al.*, 1998). In this study we have examined both a class 1 *(e1368)* and a class 2 *(e1370)* allele of *daf-2* for molecular heterogeneity over lifespan between individual nematodes.

We have used three independent and complementary techniques to examine pathophysiology of aging and age-related molecular heterogeneity in wild-type and long-lived individual nematodes. First, via 4′,6′-diamidino-2-phenylindole hydrochloride (DAPI) staining of DNA, we observed the systematic loss of nuclei from the tails of individual nematodes. This loss also occurs, but is delayed, in long-lived mutants. This approach also identified the accumulation of abnormal pathological masses of DNA in the uterus of wild-type nematodes with age. These masses are absent or much reduced in long-lived strains. Second, we detected incorporation of the nucleotide analogue 5-bromo-2-deoxyuridine (BrdU) into the observed DNA masses, demonstrating active replication of DNA in these age-related masses that can expand to occupy the entire diameter of the worm. Third, we measured the copy number of the nuclear genome in individual nematodes of various ages by real-time quantitative polymerase chain reaction (qPCR). We identified a striking heterogeneity among middle-aged nematodes with respect to nuclear genome copy number, as well as a systematic loss of nuclear genome copy number with age in both wild-type and long-lived nematodes. This is the first demonstration of genomic and nuclear variability developing with age between genetically identical individuals.

## Results

### Nuclei are systematically lost with age in wild-type and long-lived *Caenorhabditis elegans*

**Fig. 1:**
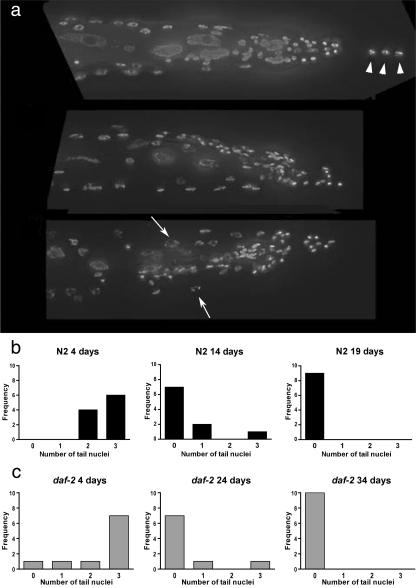
Loss of hypodermal nuclei with age from the tails of N2 and *daf-2* nematodes. (a) Tails of N2 nematodes, stained with DAPI. Worms were picked from a synchronized population initiated with a timed lay. The date of the lay is counted as day 0. Images were examined and scored as to how many nuclei were visible in the tail. In the majority of aged nematodes, the nuclei corresponding to hypodermal nuclei H9 and H10 are lost [(arrowheads, quantified in (b) and (c)]. Although not quantified, we noted the appearance of nuclei with a fragmented or condensed appearance with age (arrows, bottom panel) (top: 4 days old, middle: 14 days old, bottom: 19 days old). (b) Count of nuclei visible in the tails of individual N2 nematodes. (c) Count of nuclei visible in the tails of individual *daf-2(e1368)* nematodes. Ten individuals were counted per group, except 19-day-old N2 for which nine were counted.

We observed nuclei in aging *C. elegans* by staining with the DNA-binding dye DAPI. The nematode's optical transparency allows the visualization of every nuclei in the intact worm. Two dramatic age-related phenomena were revealed by DAPI staining. First, DAPI staining of three nuclei in the tail is lost with age (Fig. 1a,b). These likely are the nuclei of the H9 and H10 hypodermal cells (H10 being binucleate), based on comparison to published maps of hypodermal nuclei (Altun & Hall, 2005). These nuclei may be lost, or alternatively, the nuclear DNA may be degraded or damaged to such an extent that DAPI staining is lost. This nuclear loss also occurred in long-lived *daf-2(e1368)* and *daf-2(e1370)* nematodes, but was delayed relative to the occurrence in N2 (Fig. 1c and data not shown).

### Masses of DNA accumulate with age in wild-type nematodes

**Fig. 2:**
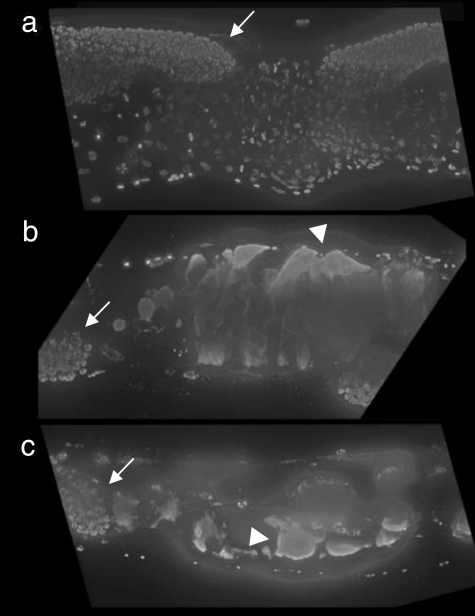
Accumulation of masses of nucleic acid in aged, wild-type N2 nematodes. (a) 4 days old. (b) 14 days old. (c) 19 days old. Worms were fixed and stained with DAPI to detect DNA. Note the mitotic nuclei at the distal tips of the gonad at each age (arrows). In the 4-day-old individual, nuclei of the developing embryos are visible. In the 14- and 19-day-old individuals, no embryos are present, and the space is filled with amorphous, tumor-like masses (arrowheads). Masses are defined as brightly staining structures not attributable to nuclei of the adult or embryos. These individuals are representative of 10 individuals of each age studied.

**Fig. 3:**
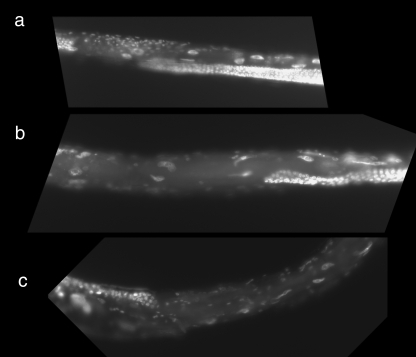
Masses of nucleic acid are not seen in *daf-2(e1370)* nematodes. (a) 4 days old. (b) 24 days old. (c) 45 days old. Worms were fixed and stained with DAPI to detect DNA. These individuals are representative of ten individuals of each age studied.

**Fig. 4:**
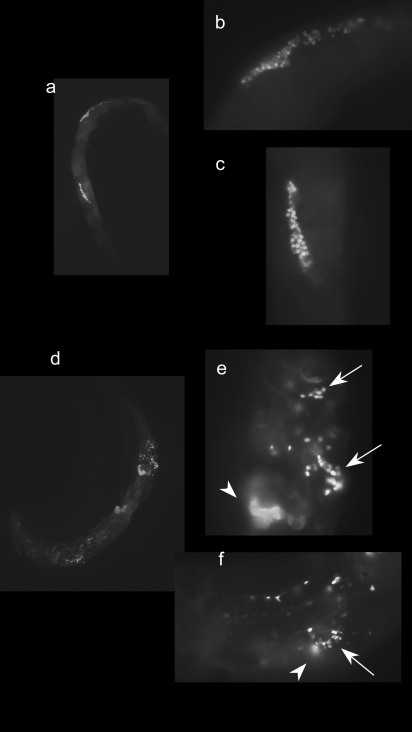
Incorporation of BrdU into the DNA of aging nematodes. N2 nematodes at 9 days of age (a,b,c) or 11 days of age (d,e,f) were exposed for 24 h to BrdU. BrdU was detected immunohistochemically using a monoclonal antibody to BrdU, and a fluorescent secondary antibody. In younger worms, incorporation of BrdU is apparent in the gonad, near the distal tips (a), and at higher magnification in (b) and (c). As worms age, label is still apparent in the gonad, although fewer nuclei are labeled, and the gonad appears disordered (d), and at higher magnification in (e) and (f) (see arrows). Also, label begins to appear in amorphous regions that are similar in appearance to those detected by DAPI staining [arrowheads in (e) and (f)]. Five of five 11-day-old animals examined displayed nongonad staining, whereas such staining was not seen in 7- or 9-day-old animals.

The second age-related phenomenon detected via DAPI staining is a massive accumulation of DAPI-stained material in the mid-section of wild-type nematodes of middle age (Fig. 2). These masses of DNA grew to essentially fill the body cavity of the nematode (Fig. 2 and Supplemental movie). Long-lived *daf-2(e1368)* nematodes (Supplementary Fig. S1) did accumulate DAPI-reactive masses, but to a lesser extent than N2 and much delayed when compared to N2. The long-lived strain *daf-2(e1370)* (Fig. 3) rarely accumulated masses. BrdU was incorporated into the region of the masses in 11-day-old N2 nematodes, but not 9-day-old N2 nematodes (Fig. 4), indicating that their generation requires active DNA synthesis that begins after 9 days of age. The acid treatment necessary for immunostaining with the anti-BrdU antibody interfered with DAPI staining of DNA, preventing costaining of the masses for BrdU and with DAPI.

### Dramatic changes in nuclear genome copy number occur with age in *Caenorhabditis elegans*

**Fig. 5:**
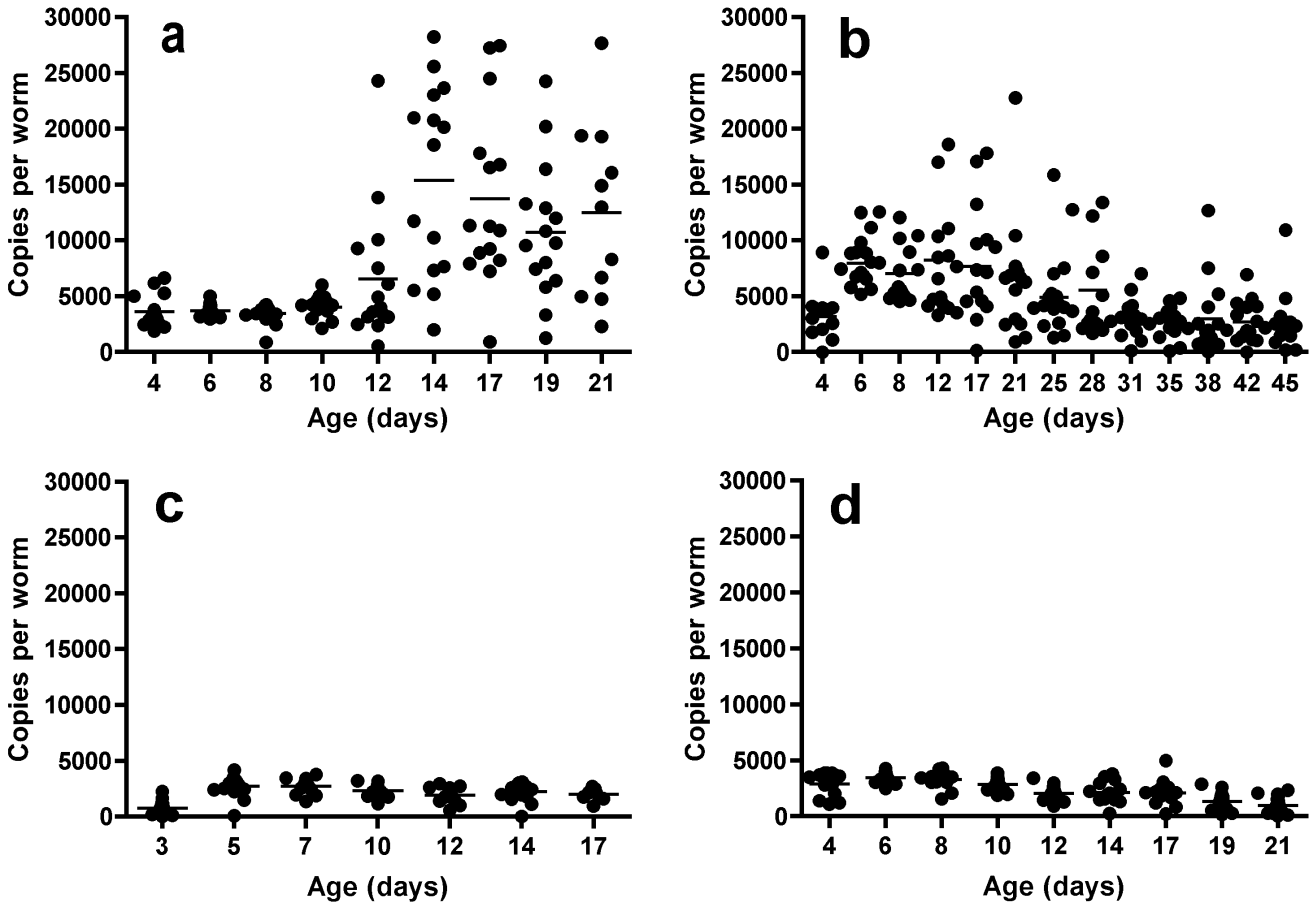
Nuclear genome copy number in individual nematodes with age. Quantitative polymerase chain reaction (qPCR) was used to quantify the number of nuclear genomes in individual nematodes. (a) N2 hermaphrodites; (b) *daf-2(e1370)* hermaphrodites, (c) N2 males, (d) N2 hermaphrodites raised on FUDR. Each symbol represents an individual nematode, 15 individuals per strain per age were assayed. The results shown are for chromosome II, but agree with results obtained for genes on chromosomes I and III (*R*^2^ = 0.96) for the same individuals.

We used quantitative real-time PCR to assay the copies of genomic DNA in individual nematodes. Consistent with the DAPI studies, in N2 worms we detected a dramatic increase in mean nuclear genome copy number at 10–12 days of age (*t*-test 4 days vs. 14 days, *P* < 0.0001) (Fig. 5a), the same point in middle age where the DNA masses were visualized. Quantitation of three single-copy genes, each located on a different *C. elegans* autosome, gave highly correlated results (*R*^2^ = 0.96, see Supplementary Fig. S2), indicating that the copy number changes involve the entire genome, and not local amplification of specific loci. The number of genomes per N2 worm was 4228 (3660 ± 1441, 4241 ± 1366, and 4784 ± 1797 for chromosomes II, III and IV, respectively) at 4 days of age, in good agreement with approximately 4000 genomes predicted from what is known about nuclear content and ploidy of young adult worms (Supplementary Table S1).

In wild-type worms, we also observed a dramatic increase in heterogeneity between individual nematodes with respect to nuclear genome copy number after 10 days of age (*P* = 0.008) (Fig. 5a). At 14 days of age, the range in genomic DNA copies is tenfold between individuals.

We also quantified the same genes in the *daf-2(e1370)* mutant via qPCR (Fig. 5b). This long-lived strain appears to largely escape the increase in nuclear genome copy number observed in wild-type worms, although interesting age-related changes in nuclear genome copy number occur in this strain as well. We observed a striking heterogeneity between individual *daf-2(e1370)* nematodes throughout the lifespan; at ages less than 12 days, *daf-2* nematodes of the same age are much more heterogeneous than N2 (two-sided *P* value from variance ratio test for all animals less than 10 days of age is 0). Interestingly, *daf-2* nematodes experience an increase in nuclear genome copy number between days 4 and 6. This may reflect an increase in body size and associated endoreduplication, or may be due to an increased amount of embryos contained in the uterus. Mean genomic copy number then falls dramatically after the age of 20 days (*t*-test 6 days vs. 45 days, *P* < 0.0001). Similar changes were observed in the strain *daf-2(e1368)* (Supplementary Fig. S3).

**Fig. 6:**
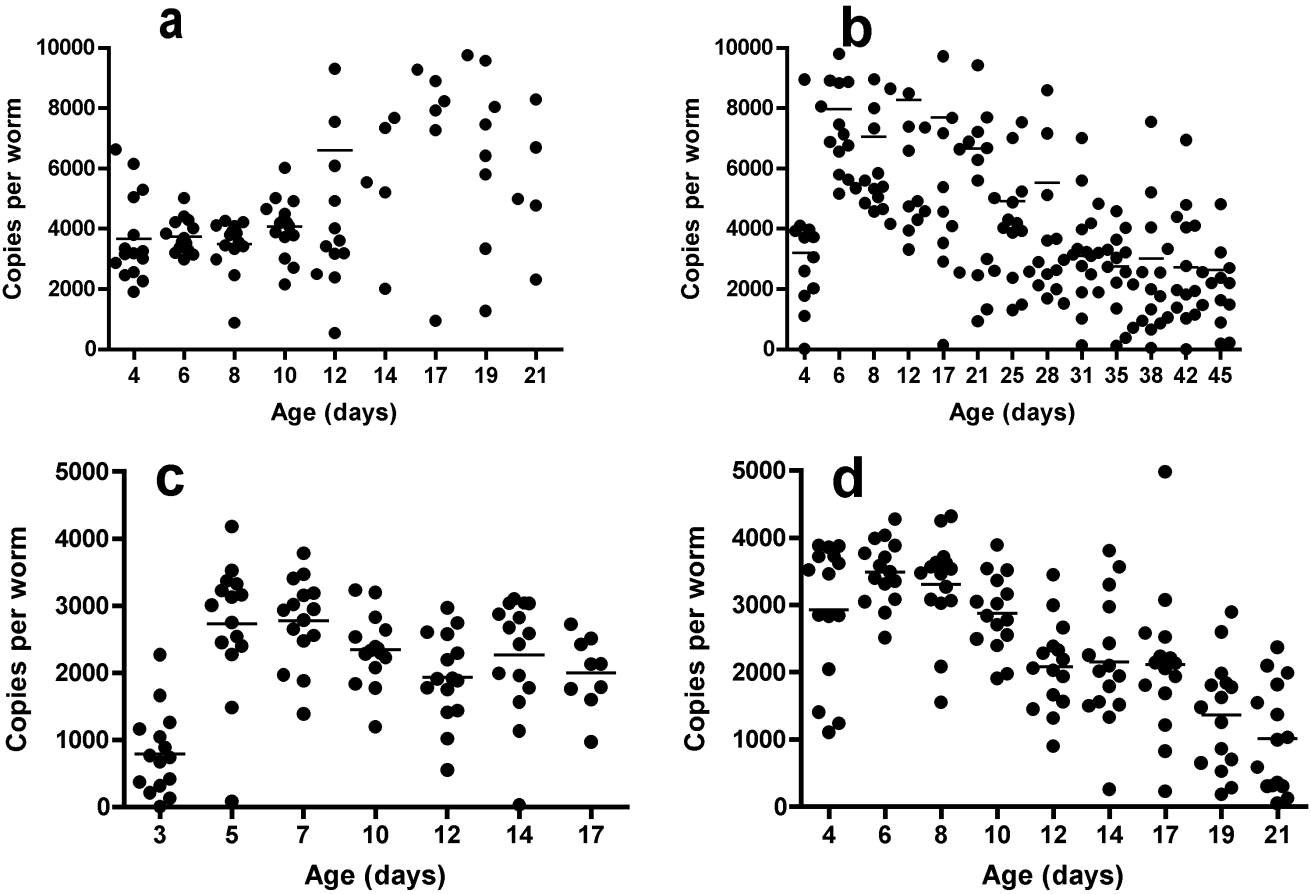
Nuclear genome copy number in individual nematodes with age. Same data as shown in Fig. 5, with an expanded scale revealing the decline in nuclear genome copy number with age in *daf-2(e1370)* hermaphrodites (b), N2 males (c) and N2 hermaphrodites raised on FUDR (d).

To verify that the accumulation of the DNA masses and increased genomic DNA copies required DNA synthesis, we grew worms in the presence of the DNA synthesis inhibitor 5-fluorodeoxyuridine (FUDR). These worms did not develop masses, and did not experience the mid-life increase in copy number (Fig. 5d). In fact, the removal of this massive mid-life proliferation of genomic DNA copies appears to have uncovered a systematic loss of genomic DNA with age (*t*-test 4 days vs. 19 days, *P* < 0.0001) (Figs 5d and 6d), to which the loss of tail nuclei we observed via DAPI staining may contribute (Fig. 1).

### The masses have an origin in the hermaphrodite germ line

To determine the cellular source of the excess genomic DNA copies, and of the DNA masses, we examined male *C. elegans*. Males did not accumulate DAPI-stained masses or experience the increase in nuclear genomes observed in N2 (Figs 5c and 6c). As a potential genetic control for the dependence of this phenomenon on oocytes, we examined the germ line proliferation mutant *glp-4*, which has few germ line nuclei at young adulthood at the restrictive temperature (Beanan & Strome, 1992). However, at the restrictive temperature almost no amplification was seen from *glp-4* DNA, and even at the permissive temperature, this strain appeared to have less genomic DNA than wild-type worms (Supplementary Fig. S4).

## Discussion

A basic, unanswered question about aging is why individuals of a homogenous population experience large differences in lifespan. Understanding whether this reflects differences in individual aging rates, or the action of different mechanisms of aging in different subsets of a population is unknown and will require the characterization of aging at the level of the individual. Only recently have studies begun to examine individual aging worms and *Drosophila*. Gene expression profiling of individual nematodes has identified age-related gene expression changes (Golden & Melov, 2004). Study of the integrity of aged worms has uncovered a stochastic element to the deterioration associated with aging (Herndon *et al.*, 2002). A study of *Drosophila* asked whether individual metabolic rates determined individual lifespan (Hulbert *et al.*, 2004). In each case, important questions about aging were examined, which can only be answered by the use of individuals. Our results begin to address the pathophysiology of aging in *C. elegans* at the molecular and cellular level*.*

We have identified dramatic changes in nuclear genome copy number and nuclear content in *C. elegans* with age. DAPI staining of DNA reveals that wild-type and long-lived strains of nematodes systematically lose three nuclei in the tail. DAPI staining also revealed an accumulation of masses of DNA in wild-type nematodes that filled the central body cavity by 14 days of age. Quantitation of the nuclear genome via quantitative real-time PCR revealed an increase in both mean copy number, as well as in intraindividual heterogeneity with respect to nuclear genome copy number, with age in wild-type *C. elegans*.

The hypodermal nuclei of the tail, H9 and H10, disappear in the majority of wild-type individuals by 14 days of age, and in *daf-2(e1368)* nematodes by 24 days of age (Fig. 1). This likely reflects cell loss with age, but may also indicate a massive amount of DNA damage or degradation preventing DAPI binding. The extent to which nuclear loss occurs in other parts of the worm is unknown at present, but this phenomenon likely contributes to the heterogeneity in nuclear DNA content we observed in middle age via qPCR (Fig. 5a). The systematic loss of nuclei with age in *C. elegans* is a novel observation; Herndon *et al.* reported necrotic death of hypodermal and intestinal nuclei, but this was occasional, stochastic and mostly end stage.

DAPI staining also detected the age-related accumulation of masses of DNA in the body cavity of wild-type nematodes (Fig. 2 and Supplemental movie). These masses appear to contain DNA that is actively replicating, as foci within the masses labeled with BrdU (Fig. 4), and the masses do not accumulate in wild-type worms grown on the DNA synthesis inhibitor FUDR (data not shown). This leads us to consider these masses to be ‘tumor-like’ growths, which represent uncontrolled DNA replication. These ‘tumors’ likely account for the heterogeneity between middle-aged and old animals with respect to DNA content, and we hypothesize may be detrimental to the health of the animals. In some animals the mass occupies so large a volume of the mid-body region that it potentially impedes the function of the intestine, which could compromise the nutritional status of aging worms.

The age-related increase in mean nuclear genome content we observed in wild-type *C. elegans* via quantitative real-time PCR (Fig. 5a) is likely substantially due to these DNA masses. The increase in nuclear genome copy number was unexpected, as *C. elegans* have ceased reproduction by 10 days of age (Johnson, 1987) and the animal is essentially post mitotic at this stage with the exception of the germ line. The increase in mean nuclear genome copy number is concurrent with the age at which BrdU was incorporated into the masses (Fig. 3), and the maximal mean copy number is achieved at the same age where the masses reach their maximal size (14 days, Fig. 2 and Supplemental movie).

The source of the massive accumulation of DNA appears to be the hermaphrodite germ line, as males do not develop the masses, and also do not demonstrate an increase in nuclear genome copy number (Fig. 5c). Growth of wild-type worms on plates containing FUDR, which results in sterility, prevents mass accumulation and the increase in nuclear genome copy number (Fig. 5d). Early studies of germ-line development and fertilization found that in mature hermaphrodites where the sperm were depleted, the nuclear membrane of unfertilized oocytes disappears, and meiosis resumes (Wood, 1988). One round of nuclear division was observed, with subsequent replication of DNA resulting in a highly polyploid oocyte. These unfertilized oocytes are normally expelled. We hypothesize that with age, these unfertilized oocytes are retained and continue to replicate, resulting in the accumulation of massive amounts of nuclear material within the animal. The long-lived *daf-2* strains did not accumulate masses to the same extent as N2 (Fig. 3 and Supplementary Fig. S1). Additionally, an age-related increase in mean genomic DNA content is not observed in the long-lived strain *daf-2(e1370)* (Fig. 5b)*.* A doubling of DNA content is observed between 4 and 6 days of age, which may reflect an increase in embryo content, or the increase in body size that occurs between these ages. After this point, however, mean genomic DNA content does not increase. This is in accord with our theory that unfertilized oocytes are a source of the masses, as *daf-2(e1370)*, which rarely developed masses, also lays almost no unfertilized oocytes (Gems *et al.*, 1998).

As an additional test of whether the hermaphrodite germ line was required for the DNA masses and changes in nuclear genome copy number, we examined the strain *glp-4*, which contains a temperature-sensitive mutation resulting in sterility and the production of only a few germ cells when grown at the restrictive temperature. We found that this strain's genomic DNA did not amplify at the restrictive temperature, giving an average genome copy number of 252 copies per worm at 4 days of age. Even when grown at the permissive temperature (Supplementary Fig. S4), the *glp-4* mutant contained less than half as many genome copies as N2 at 5 days of age, as measured by qPCR. This leads us to hypothesize that the mutation in this strain, which has not been mapped, causes some type of DNA damage that interferes with the quantitative PCR.

The biological significance of the DNA masses is unknown; these masses are so large that they might be expected to impact the health of the worm. Additionally, the synthesis of the DNA in the masses must represent a substantial allocation of resources. For these reasons, we suspect that the masses should be considered as an age-related pathology that might contribute to morbidity or even mortality of the worm. In this regard, it is interesting that the longer-lived *daf-2* strains do not experience the same proliferation of DNA. However, FUDR exposure, which prevents mass accumulation, does not extend lifespan in *C. elegans* (Gandhi *et al.*, 1980). This may be analogous to the situation that would exist if cancer were removed from the human population, which has been argued would have only a minor impact on expected mean lifespan (Olshansky *et al.*, 1990). Removal of one cause of death from a population does not necessarily change the rate of aging (i.e., the increase in the probability of death with time); death, *per se*, is perhaps not the best metric for the study of aging.

As they age, both wild-type and long-lived strains of *C. elegans* appear to lose the tight regulation held over nuclear DNA copy number apparent in young adults (Fig. 5a,b). In N2, the variance in genome content reaches a maximum as the mortality rate of a population begins to increase, and correlates with the appearance of DNA masses containing actively replicating DNA, as well as the loss of some nuclei. Individuals of the long-lived strain *daf-2(e1370)* are more heterogeneous than N2 at ages less than 14 days. This may reflect differences in nuclear content or size in the mutant strain, and suggests that characterization of the development and nuclear content of this mutant, which is so important to aging research, should be conducted. After 17 days of age, the nuclear content of *daf-2(e1370)* falls to 4-day-old levels (Fig. 5b), and a similar decline is seen in *daf-2(e1368)* (Supplementary Fig. S3b). A decline in nuclear genome copy number with age is not seen in N2 hermaphrodites, likely because the effect is swamped by the accumulation of large DNA masses in the body cavity of aging worms. However, an age-related decline is observed in N2 hermaphrodites raised on FUDR (Fig. 5d) and N2 males (Fig. 5c). This loss in nuclear genome content may reflect loss of nuclei, such as the loss of tail nuclei we detected via DAPI staining (Figs 2 and 3). It may also reflect the accumulation of DNA damage with age. Alternatively, it may result from animals with the highest genome content dying first. This could be consistent with hypotheses related to the relative fitness of the ‘oldest old’ of a population (Perls, 2002). These theories predict that the most-fit individuals remain at the end of the maximum lifespan of an organism, the weak having succumbed to disease, trauma or stochastic events. In the case of a population of *C. elegans* in which all individuals are genetically identical, the oldest old may be those who escape detrimental stochastic insults. The decay of functions other than genome maintenance would cause aging in this remaining population; evolutionary theory predicts that multiple interacting factors are responsible for the aging phenotype (Finch & Kirkwood, 2000). As they age, individual *C. elegans* have recently been observed to develop morphologic differences in a stochastic fashion consistent with the data presented here (Herndon *et al.*, 2002). These data indicate that although single-gene effects may significantly affect lifespan (Friedman & Johnson, 1988a; Guarente & Kenyon, 2000), molecular stochastic processes also have to be taken into account as significant factors that limit lifespan in individual animals.

In conclusion, we have described striking age-related changes in nuclear maintenance and morphology, and in nuclear genome maintenance in the nematode *C. elegans*. Certain nuclei are systematically lost from the tail with age, and this loss is delayed in the long-lived strain *daf-2(e1368)* and *daf-2(e1370).* Nuclear genome copy number declines dramatically in N2 males with age, and also in *daf-2(e1370),* which may reflect this nuclear loss. The fact that the loss is delayed in *daf-2* worms suggests that maintenance of nuclear integrity is associated with the *daf-2* longevity.

## Experimental procedures

### Strains

Strains used were wild-type Bristol N2, *daf-2(e1370), daf-2(e1368)*, and *glp-4(bn2ts).* The nematode strains and the *Escherichia coli* strain OP50 used in this work were provided by the Caenorhabditis Genetics Center, which is funded by the National Institutes of Health National Center for Research Resources (NCRR).

### Culture of nematodes

Nematodes were cultured at 20 °C on nematode growth media (NGM) plates, and fed *E. coli* strain OP50 (Wood, 1988). Synchronized populations were obtained by conducting a timed lay in which gravid adults were placed on a plate and removed after being allowed to lay eggs for 2–4 h. The resulting eggs were allowed to hatch and develop for 3 days, during which time the progeny were moved to new plates for survival experiments. Worms in survival experiments were moved to fresh plates at least every other day to avoid contamination by young. For culture in the presence of FUDR, worms were transferred to NGM plates containing 0.1 mg mL^−1^ FUDR at 3 days of age.

### Production of males

To generate a population of males for qPCR, N2 L4 hermaphrodites were incubated at 30 °C for 4–6 h and then returned to 20 °C. Resulting male progeny were placed onto NGM plates with L4 hermaphrodites at a ratio of 1 : 10 (hermaphrodite : males) for mating overnight at 20 °C. Males were removed, and mated hermaphrodites allowed to lay eggs on NGM plates for 4 h at 20 °C. On day 3, males were removed from lay plates and transferred to fresh NGM plates seeded with OP50 for survival analysis at 20 °C.

### Worm lysis

For the qPCR experiments shown in Supplementary Fig. S2, individual worms were picked from the plate into a PCR tube containing 5 µL of water, and frozen immediately on dry ice. Two-x lysis buffer was added to result in a final concentration of 10 mm Tris-HCl pH 8.3, 20 mm KCl, 150 µg mL^−1^ proteinase K. Lysis was carried out by incubation at 60 °C for 1 h and subsequent inactivation of the proteinase K by incubating at 99 °C for 10 min.

### Quantitative real-time PCR

#### Primer design and optimization

Primers were designed using AcePrimer (http://elegans.bcgsc.bc.ca/gko/aceprimer.shtml) and the following settings: [primer sets = 5; product size = 100 ± 50; primer size = 20 ± 2; temperature = 60 ± 2; GC clamp OFF; Nested primer interval OFF; Q value = 0.5; Primers in nearby gene ON; e-PCR word size = 7 (default); e-PCR allowed mismatches = 3; poison primers = none]. Initially, primers were designed for all five chromosomes, using the following target gene sequences: Chromosome I = smd-1 (F47G4.7), Chromosome II = cct-1 (T05C12.7), Chromosome III = ced-7 (C48B4.4), Chromosome IV = *fat-3* (W08D2.4), Chromosome V = her-1 (ZK287.8). Five primer sets were generated, as follows:

1. F tcgctcgatgatgaatcttg I.R cctgtggtcctggtcctcta
2. F caagggaccgaaatctcgta II.R cagagtgagtcgtgaaccga
3. F aagcagcaggactcacgaat III.R tgcacatgtcgttatggctt
4. F atccaatacaggtcgatggc IV.R cagctcctcctggatgtttc
5. F ttatgacagggatccgaagc V.R aaaaacgctcaccatccaag

Primer pairs were optimized by performing PCR on a serial dilution of bulk worm extract in order to determine primer efficiency, resulting in the elimination of primer pairs for chromosomes I and V and the validation of primer pairs for chromosomes II, III, and IV with efficiencies not significantly different from 100%. These primer pairs were used in all subsequent studies.

#### PCR conditions

PCR reactions (10 µL per well) contained 5 µL worm extract (equivalent to 1/30 of an extract from a single worm), 500 nm forward and reverse primers, and 1× SYBR Master Mix (Applied Biosystems, Foster City, CA, USA). Reactions were set up in a larger volume (2.5× volume = 25 µL total) and aliquoted into duplicate 10 µL wells for real-time amplification. Cycling conditions were: 95 °C × 10 min to activate hot start Taq polymerase followed by 40 cycles of 95 °C × 15 s + 60 °C × 1 min, followed by a dissociation (melt) stage ramping from 60 °C to 95 °C at 2% of maximal ramp rate. Cycling was achieved in 384 well plates on a 7900 HT Sequence Detection System (Applied Biosystems), and fluorescence intensity data was collected at the annealing stage (60 °C) and during dissociation. Raw data were converted to cycle threshold (Ct) values using SDS version 2.1.1 software (Applied Biosystems), and relative quantities calculated using the delta Ct method. For example, for the N2 hermaphrodites, there were a total of 9 time points × 15 worms per time point = 135 samples × 2 wells per sample = 270 wells per chromosome primer pair, that were run on a single 384 well PCR plate. Hence for these samples, a total of three 384 well plates were run, one for each primer pair.

#### Standards and quantitation

All extracts for a given strain were run on a single 384 well PCR plate, with the exception of *daf-2*, which was split onto two plates (as there were a total of 13 × 15 × 2 = 390 wells required). In order to ensure that there was no plate-to-plate variation in PCR amplification efficiency, PCR run plates contained 22 replicate wells of a master extract of a pool of N2 worms, for comparison across plates run at different times. There were no significant differences between plates run using a given primer pair in the mean Ct of these positive controls. All plates also contained no-template control wells, none of which amplified. Melt curve analysis was used to ensure that amplified wells contained actual amplicons, as opposed to primer dimers (which were very rare). Conversion of Ct values into copy number was performed using dsDNA standards (PCR amplicons, purified and quantitated using Picogreen dye and the calculated molecular weight for the length of the dsDNA product). From each strain assayed, 15 samples (calibrators) from the youngest age animals were amplified together a second time on a plate containing a standard curve of known copy number of dsDNA standards. Cycle thresholds from these runs were used to calculate copy numbers for all 15 calibrators, which were then used to convert prior Ct values into copy numbers. These quantifications were performed independently for each set of primer pairs.

### Statistical analysis

To compare mean nuclear genome content between two groups of individuals, we carried out an unpaired *t*-test.

We tested the trend in variance with age by simply (i) calculating the sample variance at each age, and (ii) regressing this sample variance vs. age. In order to derive robust inference, we used a permutation test (using the typical Z-statistic on the slope of each regression) to derive exact *P* values.

For testing the equivalence of a variance at a set of time point (i.e., between two different strains), we used a variance ratio test (Pagano & Gauvreau, 1993).

### DAPI staining

Nematodes were fixed overnight in Carnoy's solution, allowed to dry on slides overnight, and stained with mounting medium containing DAPI. Imaging was conducted on a Zeiss Axioplan 2 microscope. We collected low-magnifcation (10×) images of each worm, and high magnification (63×) optical sections of the middle and the tail of each worm. Images were deconvoluted using deconvolution software from either Huygens scientific or AutoDeblur (AutoQuant). Both deconvolution algorithms significantly improved the images over the raw images acquired, whereas maintaining image detail. After deconvolution, tail image stacks were assembled into a three-dimensional image using a combination of Autodeblur and Aftereffects (Adobe).

### BrdU labeling

N2 nematodes at 7, 9, and 11 days of age were exposed to BrdU by adding 0.5 mL of a 10-mm solution of BrdU in S medium to 6 cm plates containing the worms. The worms were left on the plates overnight, collected, rinsed, and fixed in 4% paraformaldehyde. After fixation, worms were permeabilized by exposure to β-mercaptoethanol followed by collagenase digestion of the cuticle. Worms were then exposed to 2N HCl for 1 h. This acid treatment was found to be necessary to allow the anti-BrdU antibody access to incorporated BrdU. However, this treatment interfered with the ability to detect DNA with DAPI or other DNA-reactive dyes, so we were unable to collect images of both DAPI-stained and BrdU-labeled DNA from the same worm. BrdU was detected with a primary monoclonal antibody (Sigma, St. Louis, MO, USA), and a fluorescent labeled goat antimouse secondary antibody (Molecular probes, Eugene, OR, USA).

## Supplementary material

The following supplementary material is available for this article:

Fig. S1 DAPI staining of long-lived daf-2(e1368) and wild type N2 nematodes. Young (4-day, top left panel) and middle-aged (24-day, top right panel) daf-2 nematodes were stained with the DNA-reactive dye DAPI. Staining reveals an accumulation of some DAPI-reactive material, similar to that seen in wild-type N2 nematodes but the accumulation of DNA is much less prevalent than in the wild-type strain (4-day, bottom left panel, and 14-day, bottom right panel).

Fig. S2 Nuclear genome copy number quantitation using three different primer sets on three different chromosomes results in highly correlated results. The quantified nuclear genome copy number for each individual nematode used in the study is plotted. (a) Chromosomes II vs. IV, (b) Chromosomes II vs. III, (c) Chromosomes IV vs. III.

Fig. S3 Two additional quantitative PCR methods reveal the same trends in nuclear genome copy number for N2 and daf-2. Quantitation of nuclear genome copy number via qPCR in individual N2 (a) and daf-2 (b) nematodes. Circles = MJ 96-well plate format (and daf-2(e1368) in b). Triangles = LightCycler (and daf-2(e1370) in b). The mean copy number of genomes per animal increases with age for N2 (t test 4 vs. 24 days, *P* < 0.0001 MJ data set, *P* = 0.0013 LightCycler data set). The mean copy number also increases for daf-2 between 4 and 19 days of age (*P* = 0.04 4 vs. 19 days, LightCycler data set, *P* = 0.001 MJ data set), and then falls with age (*P* < 0.0001 19 vs. 59 days, LightCycler data for daf-2(e1370), *P* = 0.056 14 vs. 34 days, MJ data for daf- 2(e1368)). See supplemental methods.

Fig. S4 The germline deficient mutant glp-4 does not have a wild-type nuclear genome content, even at the permissive temperature. The number of copies of the nuclear genome, as assayed by qPCR, for individual N2 or glp-4 nematodes at either 25 °C or 17 °C are shown. Each dot represents a single worm, and 15 individuals were assayed per condition.

## Supplemental Table I: predicted nuclear genome copy number in young adult N2 hermaphrodites

Table S1 Predicted nuclear genome copy number in young adult N2 hermaphrodites.

Movie S1 DAPI staining reveals masses of DNA in the region of the uterus in aged worms. These masses become apparent in middle age, and by 14 days of age, can fill much of the internal space of the worm. In this movie clip, the extent of the DAPIstained masses in a characteristic 14-day-old wild-type nematode is visualized in three dimensions. Serial optical sections were collected at 63x magnification (see methods). The left panel shows a reconstruction of the worm in three dimensions, while in the right panel the optical sections are displayed in succession as the focal plane moves through the worm.

This material is available as part of the online article from:

http://www.blackwell-synergy.com/doi/abs/10.1111/j.1474-9726.2007.00273.x (This link will take you to the article abstract).

## References

[b1] Altun ZF, Hall DH (2005). Epithelial system. Atlas of *C. elegans* anatomy – an illustrated handbook; Chapter 2: Adult organs and tissues.

[b2] Apfeld J, Kenyon C (1999). Regulation of lifespan by sensory perception in *Caenorhabditis elegans*. Nature.

[b3] Beanan MJ, Strome S (1992). Characterization of a germ-line proliferation mutation in *C. elegans*. Development.

[b4] Finch CE (1990). Longevity, Senescence, and the Genome.

[b5] Finch CE, Kirkwood TBL (2000). Chance, Development, and Aging..

[b6] Friedman DB, Johnson TE (1988a). A mutation in the *age-1* gene in *Caenorhabditis elegans* lengthens life and reduces hermaphrodite fertility. Genetics.

[b7] Friedman DB, Johnson TE (1988b). Three mutants that extend both mean and maximum life span of the nematode, *Caenorhabditis elegans*, define the *age-1* gene. J. Gerontol. Biol. Sci..

[b8] Gandhi S, Santelli J, Mitchell DH, Stiles JW, Sanadi DR (1980). A simple method for maintaining large, aging populations of *Caenorhabditis elegans*. Mech. Ageing Dev..

[b9] Garigan D, Hsu AL, Fraser AG, Kamath RS, Ahringer J, Kenyon C (2002). Genetic Analysis of Tissue Aging in *Caenorhabditis elegans*. A role for heat-shock factor and bacterial proliferation. Genetics.

[b10] Gems D, Sutton AJ, Sundermeyer ML, Albert PS, King KV, Edgley ML, Larsen PL, Riddle DL (1998). Two pleiotropic classes of *daf-2* mutation affect larval arrest, adult behavior, reproduction and longevity in *Caenorhabditis elegans*. Genetics.

[b11] Golden TR, Melov S (2004). Microarray analysis of gene expression with age in individual nematodes. Aging Cell.

[b12] Guarente L, Kenyon C (2000). Genetic pathways that regulate ageing in model organisms. Nature.

[b13] Herndon LA, Schmeissner PJ, Dudaronek JM, Brown PA, Listner KM, Sakano Y, Paupard MC, Hall DH, Driscoll M (2002). Stochastic and genetic factors influence tissue-specific decline in ageing *C. elegans*. Nature.

[b14] Hulbert AJ, Clancy DJ, Mair W, Braeckman BP, Gems D, Partridge L (2004). Metabolic rate is not reduced by dietary-restriction or by lowered insulin/IGF-1 signalling and is not correlated with individual lifespan in *Drosophila melanogaster*. Exp. Gerontol..

[b15] Johnson TE (1987). Aging can be genetically dissected into component processes using long-lived lines of *Caenorhabditis elegans*. Proc. Natl Acad. Sci. USA.

[b16] Johnson TE (1990). Increased life-span of *age-1* mutants in *Caenorhabditis elegans* and lower Gompertz rate of aging. Science.

[b17] Kenyon C, Chang J, Gensch E, Rudner A, Tabtiang R (1993). A *C. elegans* mutant that lives twice as long as wild type [see comments]. Nature.

[b18] Kimura KD, Tissenbaum HA, Liu Y, Ruvkun G (1997). *daf-2*, an insulin receptor-like gene that regulates longevity and diapause in *Caenorhabditis elegans*[see comments]. Science.

[b19] Lin K, Dorman JB, Rodan A, Kenyon C (1997). *daf-16*: an HNF-3/forkhead family member that can function to double the life-span of *Caenorhabditis elegans*[see comments]. Science.

[b20] Ogg S, Paradis S, Gottlieb S, Patterson GI, Lee L, Tissenbaum HA, Ruvkun G (1997). The Fork head transcription factor DAF-16 transduces insulin-like metabolic and longevity signals in *C. elegans*. Nature.

[b21] Olshansky SJ, Carnes BA, Cassel C (1990). In search of Methuselah: estimating the upper limits to human longevity. Science.

[b22] Pagano M, Gauvreau K (1993). Principles of Biostatistics..

[b23] Perls T (2002). Genetic and environmental influences on exceptional longevity and the AGE nomogram. Ann. N Y Acad. Sci..

[b24] Wood WB (1988). The Nematode Caenorhabditis elegans.

