# Supplementary material for: Dramatic age-related changes in nuclear and genome copy number in the nematode Caenorhabditis elegans
Source: Aging Cell. 2007 Apr 1;6(2):179–88. doi: 10.1111/j.1474-9726.2007.00273.x (PMC2049047; doi:10.1111/j.1474-9726.2007.00273.x)
Supplement: Fig. S1 — DAPI staining of long-lived daf-2(e1368) and wild type N2 nematodes. Young (4-day, top left panel) and middle-aged (24-day, top right panel) daf-2 nematodes were stained with the DNA-reactive dye DAPI. Staining reveals an accumulation of some DAPI-reactive material, similar to that seen in wild-type N2 nematodes but the accumulation of DNA is much less prevalent than in the wild-type strain (4-day, bottom left panel, and 14-day, bottom right panel). [file ace0006-0179-fs1.doc]

**Supplemental material**

**Supplemental Figures**

**Supplemental Figure 1****.**


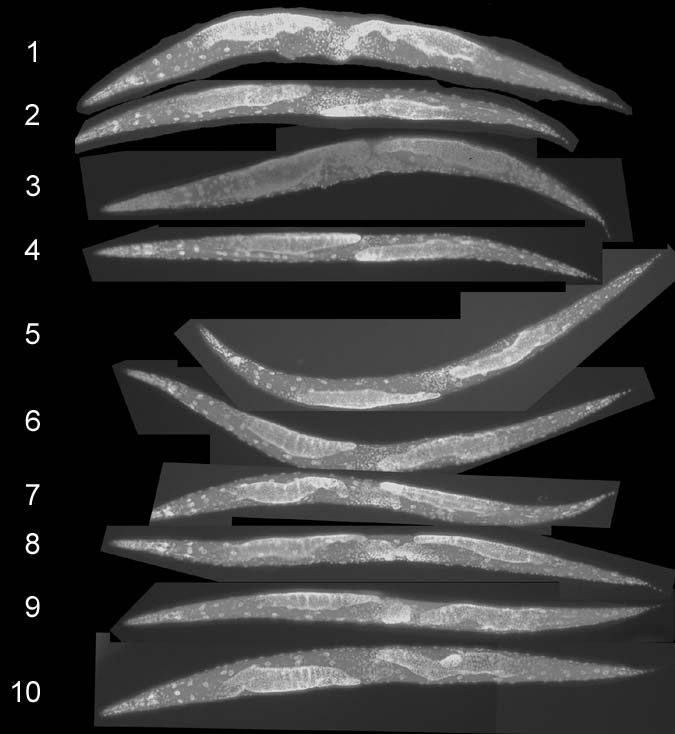

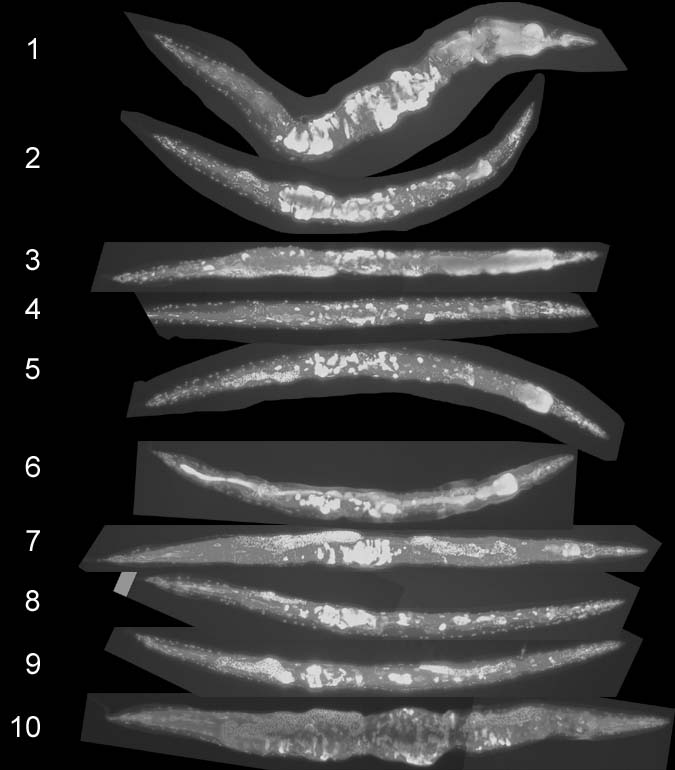


**Supplemental Figure 2.**

**Supplemental Figure 3.**

**
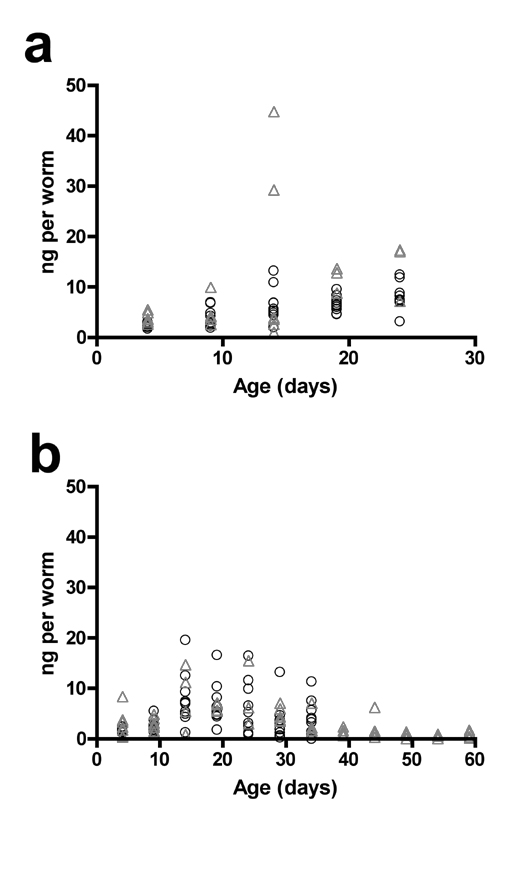
**

**Supplemental Figure 4.**


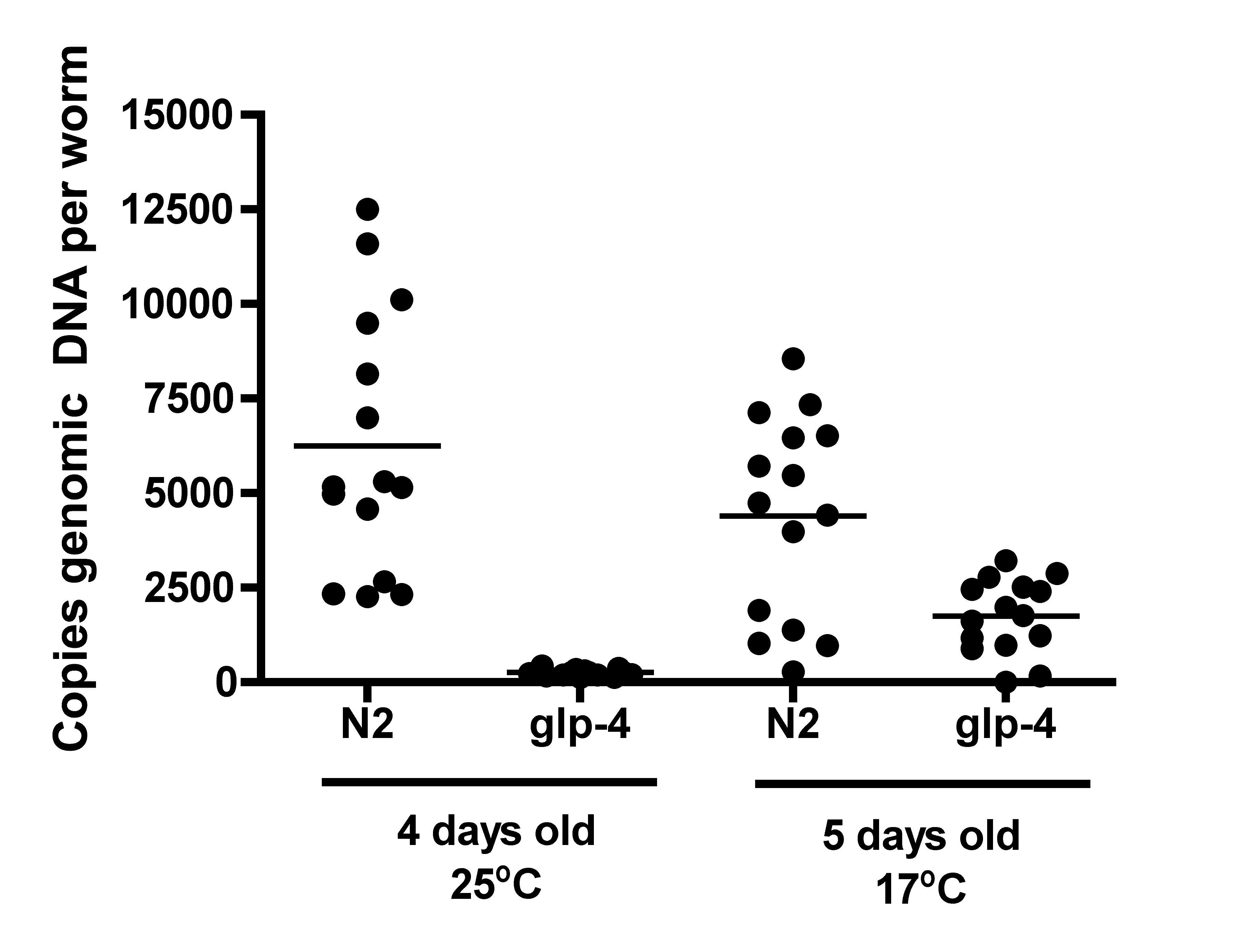


**Supplemental Table I**

Nuclear ploidy is based upon studies using Feulgen or Hoechst 33258 dyes, summarized in Wood (1988) pg. 49.

**Supplemental Methods**

**Worm Lysis**: For the qPCR experiments shown in supplemental figure 2, individual worms were picked from the plate into a PCR tube containing 10µl of lysis buffer (50mM Tris-HCl pH 8.3, 20mM KCl, 150µg/ml proteinase K). Lysis was carried out by freezing the tube containing an individual nematode in lysis buffer at –80^o^C for 10 min, followed by incubation at 60^o^C for 1 hour and subsequent the proteinase K treatment at 99^o^C for 10 minutes.

**Quantitative real-time PCR**: Quantitative real-time PCR was carried out in an Opticon (MJ Research) or in a Light-cycler (Roche) in which accumulation of PCR product is followed by measuring the increase in fluorescence of the double-stranded DNA-binding dye, SYBR green. Three single-copy genes located on different *C. elegans* chromosomes were assayed: the S-adenosylmethionine decarboxylase gene (SAMDC){Da'dara, 1998 #84} , the chaperonin subunit 1 gene (cct-1){Leroux, 1995 #176}, the ced-7 gene, and the her1 gene. The primer pairs used for nuclear copy number estimates were:

Forward reverse

samdc5 TGAATGACACTGACCAATAC samdc6 ACCTGAAACACTGAAGAAC

cct1-1 TCACAAATGACGGAGCCAC cct1-2 CGCCACAATAACGACAGAAG

ced7-1 ca cagtcagtaggtatccagT ced7-2 AGAGAGAAAGCCAACAGAA

her1-1 tctattcctcattacacctccc her1-2 gcaactcgacacacatgac.

PCR reactions contained the following: 50 mM Tris-HCl (pH 8.0), 20 mM KCl, 500 µg/ml BSA, 0.8 U Taq (Roche), 1 X SYBR green (Molecular Probes), 0.2 mM dNTPs, 0.5 mM each primer, and either 5 mM MgCl_2_ (SAMDC reactions) or 4 mM MgCl_2_ (all other primer pairs) in a 20 μl reaction. 10X SYBR green was a 1:1000 dilution of the 10,000X stock from Molecular Probes.

Opticon cycling parameters were different for each primer pair but all included 3 steps: (1) denaturation at 95^o^C for 30 sec, (2) 45 cycles of PCR followed by monitoring the fluorescence of SYBR green, (3) melting curve analysis to ensure specific product quantification.

Light-Cycler cycling parameters were different for each primer pair but all included 3 steps: (1) denaturation at 95^o^C for 30 sec, (2) 45 cycles of PCR followed by monitoring the fluorescence of SYBR green, (3) melting curve analysis to ensure specific product quantification . For SAMDC, the PCR cycling conditions were: denaturation at 95^o^C for 0 sec, annealing at 57^o^C for 0 sec, extension at 72^o^C for 1 sec followed by fluorescence detection at 84^o^C. For cct-1, the PCR cycling conditions were: denaturation at 95^o^C for 0 sec, annealing at 61^o^C for 0 sec, extension at 72^o^C for 1 sec followed by fluorescence detection at 82^o^C. PCR cycling conditions for mitochondrial primers 9b and 6 were: denaturation at 95^o^C for 0 sec, annealing at 61^o^C for 0 sec, extension at 72^o^C for 1 sec followed by fluorescence detection at 76^o^C. PCR cycling conditions for mitochondrial primers 3 and 4 were: denaturation at 95^o^C for 0 sec, annealing at 60^o^C for 0 sec, extension at 72^o^C for 1 sec followed by fluorescence detection at 74^o^C. All temperature transitions were at 20 ^o^C/sec except for the melting analysis. Quantitation of genome copy numbers in worm extracts was carried out with the LightCycler software based on standard curves run with each analysis, and using the fit points method and the arithmetic background adjustment provided by the software. Replicate assays of the same worm lysate were within 15% of each other.
